# Genome-scale measurement of off-target activity using Cas9 toxicity in high-throughput screens

**DOI:** 10.1038/ncomms15178

**Published:** 2017-05-05

**Authors:** David W. Morgens, Michael Wainberg, Evan A. Boyle, Oana Ursu, Carlos L. Araya, C. Kimberly Tsui, Michael S. Haney, Gaelen T. Hess, Kyuho Han, Edwin E. Jeng, Amy Li, Michael P. Snyder, William J. Greenleaf, Anshul Kundaje, Michael C. Bassik

**Affiliations:** 1 Department of Genetics, Stanford University, Stanford, California 94305, USA; 2 Department of Computer Science, Stanford University, Stanford, California 94305, USA; 3 Program in Cancer Biology, Stanford University, Stanford, California 94305, USA; 4 Stanford University Chemistry, Engineering, and Medicine for Human Health (ChEM-H), Stanford, California 94305, USA

## Abstract

CRISPR-Cas9 screens are powerful tools for high-throughput interrogation of genome function, but can be confounded by nuclease-induced toxicity at both on- and off-target sites, likely due to DNA damage. Here, to test potential solutions to this issue, we design and analyse a CRISPR-Cas9 library with 10 variable-length guides per gene and thousands of negative controls targeting non-functional, non-genic regions (termed safe-targeting guides), in addition to non-targeting controls. We find this library has excellent performance in identifying genes affecting growth and sensitivity to the ricin toxin. The safe-targeting guides allow for proper control of toxicity from on-target DNA damage. Using this toxicity as a proxy to measure off-target cutting, we demonstrate with tens of thousands of guides both the nucleotide position-dependent sensitivity to single mismatches and the reduction of off-target cutting using truncated guides. Our results demonstrate a simple strategy for high-throughput evaluation of target specificity and nuclease toxicity in Cas9 screens.

## Abstract (web summary)

CRISPR-Cas9 screens are powerful high-throughput tools but can be confounded by nuclease toxicity. Here the authors design a library of variable length gRNAs with thousands of negative controls, including the targeting of ‘safe' loci to account for on-target site DNA damage toxicity.

Genome-wide screens using the CRISPR-Cas9 system have been highly effective for determination of gene function^1^,^2^,^3^,^4^,^5^. While earlier RNA interference-based screening technologies have been highly effective^6^,^7^,^8^,^9^, they can suffer from low on-target efficacy, non-specific toxicity, and pervasive off-target effects^10^,^11^,^12^,^13^,^14^,^15^,^16^. The extent to which similar flaws also exist in Cas9 screens is under active investigation. Cas9 on-target efficacy is high, but the existence of in-frame indels can limit efficacy, as has been observed in large-scale screens^1^,^10^,^17^,^18^,^19^. The existence of non-specific toxicity resulting from Cas9 expression or nuclease activity has been previously proposed^20^,^21^ and more recently direct evidence has been found^22^,^23^ suggesting that this toxicity generates false-positives in screens for essential genes. Finally, although Cas9 off-target activity has been extensively investigated^24^,^25^,^26^,^27^,^28^,^29^,^30^,^31^,^32^, it remains unresolved whether off-target effects confound results from large-scale screens.

Non-specific toxicity of reagents can affect interpretation of high-throughput screens. For example, shRNA overexpression can cause toxicity via misregulation of the endogenous miRNA processing machinery^12^. Non-targeting shRNAs have been used as negative controls to account for these effects, allowing accurate modelling of the null distribution and accurate hit calling^15^,^16^,^33^. Similarly, studies using Cas9 have included non-targeting single-guide RNAs (sgRNAs)^22^,^23^,^34^,^35^,^36^, which are overexpressed and loaded into Cas9, presumably controlling for the potentially disruptive binding of Cas9 to PAM sites throughout the genome^30^,^37^. However these non-targeting sgRNAs may fail to replicate the most dramatic, non-specific effect of Cas9 gene knockouts: the formation of double-strand breaks in genomic DNA^22^,^23^. In fact, cutting at amplified regions—where a single cut site results in numerous double-strand DNA breaks—has been found to be toxic across numerous cell lines^22^,^23^,^36^. Similarly, guides with large numbers of target sites have also been found to be toxic^38^.

Numerous strategies for reducing Cas9 off-target effects have been developed^31^, including paired nickases^39^, truncated guides^32^,^40^, FOKI dimer fusions^41^, and modifications to Cas9 itself^42^,^43^. Assays for genome-wide double-strand DNA breaks^26^,^27^,^28^,^32^ have indicated these strategies successfully limit off-target cutting. However, these experiments have so far been limited to measurement of the off-target cutting of a handful of guides, leaving open the question of how these off-targets may interfere with the output of high-throughput screens, and if the varied strategies for off-target reduction can be effective in this domain.

The use of truncated guides of length 17–18 bp has shown great promise in both reduction of off-targets and preservation of on-target activity^32^,^40^. Based on both low-throughput sequencing of candidate off-target sites^40^ and high-throughput determination of off-targets with GUIDE-seq for a handful of guides^32^, truncated guides appear to have fewer off-targets. Though reduced overall activity of truncated sgRNAs could be responsible for this reduction in off-target activity, low-throughput tests suggest that this is not the case in either human cell lines^40^ or yeast^44^.

Here we present results from a novel genome-wide CRISPR-Cas9 deletion library in three cell lines. We demonstrate the existence of non-specific toxic effects from cutting on- and off-target sites and design a strategy to control for them. We take advantage of this toxicity to assay thousands of guides for off-target activity using growth as a phenotype. Using this system we can extract generalizable conclusions about off-target activity and provide evidence that truncated sgRNAs^40^ can improve specificity with little detectable loss in on-target activity in high-throughput screens.

## Results

### CRISPR deletion library

**Figure 1:**
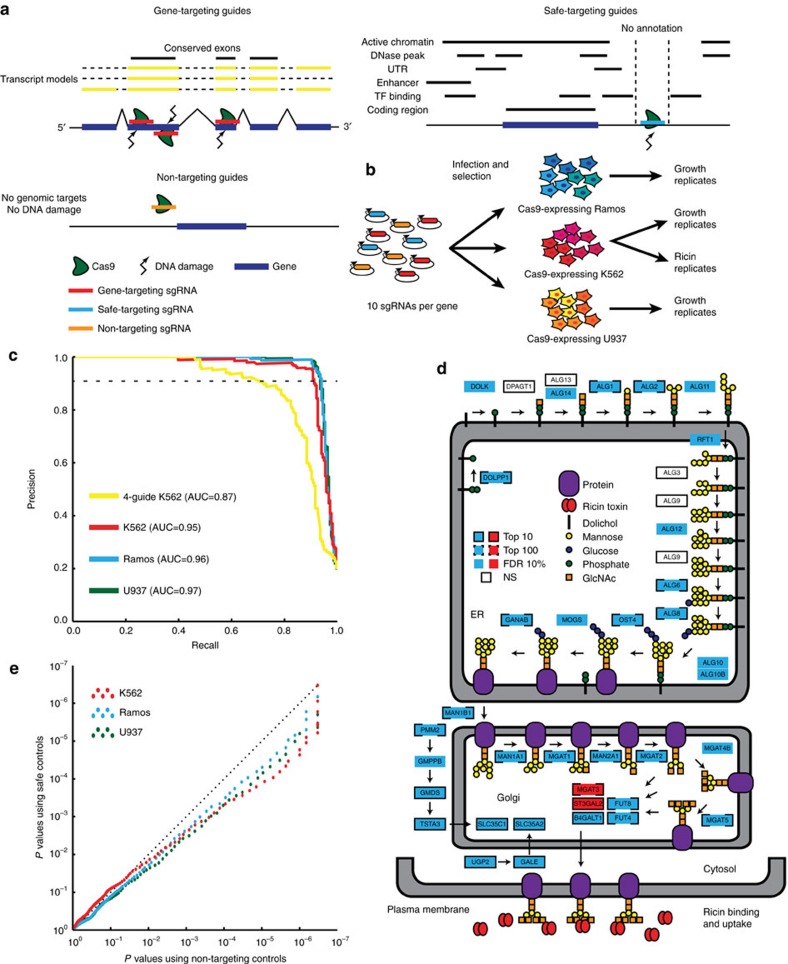
Design and performance of Cas9 library. (**a**) Schematic of library design indicating design of gene-targeting guides, safe-targeting guides, and non-targeting guides. (**b**) Growth screens were performed in duplicate in three cell lines, and a ricin screen was performed in duplicate in a single cell line. (**c**) Precision/recall curve for performance on gold-standard essential genes^38^. These curves graphically display the trade-off between the fraction of genes correctly identified as essential (precision) and the fraction of all essential genes identified (recall). Untreated conditions were compared to plasmid library composition and replicates were combined and analysed with casTLE^20^. Previous 4-guide duplicate screen in K562 included as reference^20^. (**d**) Schematic of nucleotide sugar and n-glycan synthesis genes in ricin screen results. Ricin treated conditions were compared to untreated conditions in K562, and replicates combined and analysed using casTLE^20^. Blue boxes indicate the gene knockout protected the cell from ricin while red boxes indicate the gene knockout sensitized the cell to ricin. NS indicates non-significance (*q*>0.1). White boxes indicate that these genes are known to be on-pathway but were not identified as ricin regulators. (**e**) Quantile-quantile plot showing altered distribution of *P* values using safe-targeting or non-targeting control in growth screens. *P* values are calculated from both replicates using casTLE^20^.

To evaluate the effects of nuclease toxicity and sgRNA length in genome-wide CRISPR screens, we designed a 10-sgRNA-per-gene CRISPR-Cas9 deletion library targeting all ∼20,500 protein-coding human genes (Fig. 1a; Supplementary Data 1; see methods for complete description). sgRNAs were chosen to balance (1) on-target potential to cause deleterious indels, as predicted by placement within the gene (Supplementary Fig. 1a), GC content (Supplementary Fig. 1b), and exon conservation (Supplementary Fig. 1c), and (2) off-target activity, as predicted by the number of 0-, 1- and 2-bp mismatch off-target sites (Supplementary Fig. 2a–c). In order to determine the effect of sgRNA length, the library was designed to contain guides ranging from 17 to 20 bp in length (Supplementary Fig. 1d). Finally, to ensure multiple regions of the same gene were targeted, guides with overlapping target sites or targeting identical exons were avoided. To monitor the effect of nuclease-dependent toxicity, two distinct classes of negative controls were included: non-targeting controls with no binding sites in the genome and safe-targeting guides targeting genomic locations with no annotated function (Fig. 1a), discussed below. For ease of use, the library is synthesized as 9 sublibraries of functionally related genes (Supplementary Fig. 3a). A library targeting all ∼23,000 protein-coding mouse genes was designed using identical rules, but split into 20 distinct sublibraries to enable *in vivo* screens (Supplementary Data 2; Supplementary Fig. 3b).

To validate the library, we infected Cas9-expressing K562, Ramos, and U937 human cell lines and grew replicate cultures to identify genes required for growth (Fig. 1b; see also Methods section). Library performance was evaluated using a previously defined set of gold-standard essential and nonessential genes^38^; these are predicted from expression or screen results to be either essential or nonessential for growth in all human cell lines. We find the results are highly reproducible (Supplementary Fig. 4a–c) and almost perfectly distinguish gold-standard essential and nonessential genes^38^ in each cell line (Fig. 1c; Supplementary Data 3 and 4). This greatly outperformed our previous Cas9 and shRNA library designs^16^,^20^,^35^, with >88% of gold-standard essential genes identified at 1% false positive rate versus the 60% identified by previous libraries.

To validate the library for screens other than growth, we performed a screen for modifiers of ricin toxicity in K562 cells in duplicate (Fig. 1b; Supplementary Fig. 4d; Supplementary Data 3 and 4), as we have extensive data on genes which modify this response^16^,^33^. While there are no gold-standard genes affecting ricin resistance, the screen with this library robustly identified genes involved in ricin resistance by several metrics. First, the screen identified known ricin regulators at 10% false discovery rate (FDR) including 32 of 48 genes validated from previous shRNA and CRISPRi screens^33^,^45^. 13 of the 16 genes not identified in this CRISPR screen are essential for growth (Supplementary Data 4) and would not be expected to be identified in a knockout screen. Beyond this, 895 genes were identified at 10% FDR, including a large number of genes which had not been previously implicated in ricin biology. Second, the newly identified genes included nearly every member of several interconnected nucleotide sugar and n-glycan synthesis pathways (Fig. 1d). These enzymes synthesize the cell surface β-linked Gal/GalNAc-containing glycans, which are bound by the ricin B-chain lectin and required for its uptake into the cell^46^,^47^. Effective identification of known and novel genes affecting ricin toxicity, as well as essential genes across three cell types, validate the presented CRISPR-Cas9 library as a robust tool for genome-wide screens.

### Modelling non-specific toxicity with safe-targeting guides

To control for the potential effects of double-strand DNA breaks, we designed a set of guides targeting non-genic regions with no annotated function across 127 cell lines (Fig. 1a; Supplementary Data 5; and Methods section). These safe regions have no active chromatin marks, no experimentally or computationally determined transcription factor binding sites, no DNase accessibility signal, and are not conserved. Safe-targeting guides induce the same genomic DNA cutting as gene-targeting guides, as well as their overexpression and loading into Cas9, and therefore should theoretically provide better controls than non-targeting guides.

In all growth screens performed, we find safe-targeting sgRNAs are more toxic than their non-targeting counterparts, as measured by a relative depletion of the safe-targeting guides at late time points (Mann–Whitney test comparing safe-targeting to non-targeting guides; *P* value<10^−26^ for all replicates; Supplementary Fig. 5). The negative growth effect of safe-targeting guides is likely due to DNA damage and the subsequent repair response^22^,^23^. How this non-specific growth effect will affect phenotypes in all non-growth screens is less clear, but in our screen for genes affecting ricin toxicity in K562 cells (Fig. 1b), the use of non-targeting controls underestimates the true background noise as modelled by a distribution of safe-targeting sgRNAs (Kolmogorov–Smirnov test comparing safe-targeting guides to non-targeting guides; *P* value<10^−7^ for both replicates; Supplementary Fig. 6a,b). To test the impact of using safe-targeting guides for hit discovery, we examined the distribution of *P* values generated from the combination of replicates using casTLE^20^ with either the non-targeting controls only or the safe-targeting controls only. We find that using the non-targeting controls results in anti-conservative *P* values, that is, *P* values are more significant than when using safe-targeting controls, in our growth screens (Fig. 1e) and both overly conservative and anti-conservative tests in our ricin screen (Supplementary Fig. 6c). Anti-conservative *P* values can lead to false-positives as genes will appear more significant than they are, and conservative tests can lead to false-negatives as genes will appear less significant. A concrete example can be seen in an analysis of K562 growth data: at a 1% FDR cutoff, ∼2,100 genes with growth defects upon deletion are identified using the non-targeting controls, while ∼1,900 genes are identified using safe-targeting controls (Supplementary Data 6). This suggests safe-targeting controls can both prevent false-positive results in growth screens as well as more accurately determine significance in non-growth screens.

### Detection of off-target toxicity

**Figure 2:**
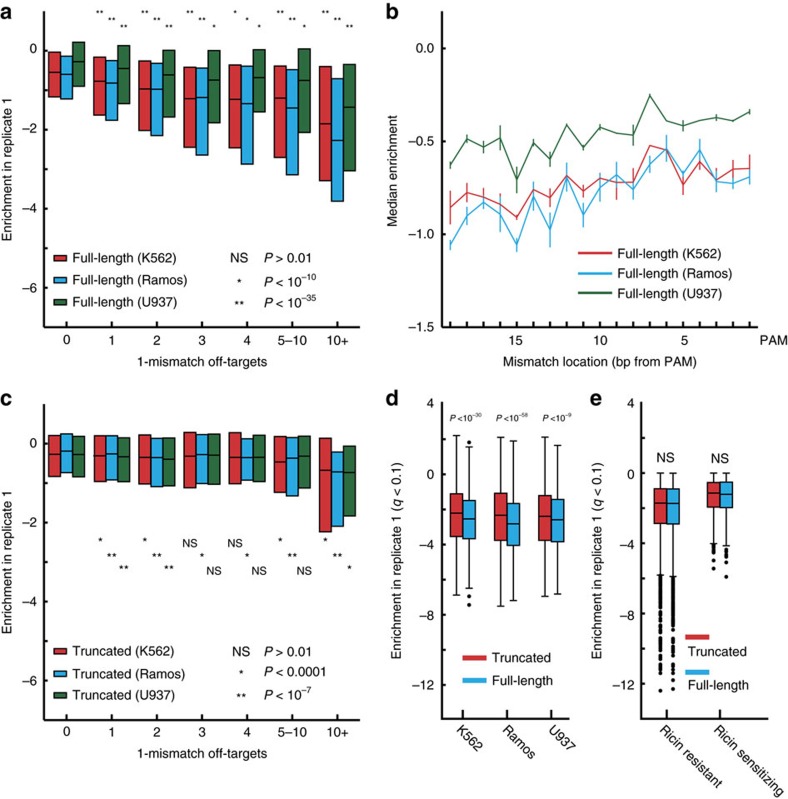
Off-target detection using nuclease-induced toxicity. (**a**) Median and quartile range of full-length 19 and 20 bp guides in three cell lines by number of 1-mismatch off-targets. *P* values are from a Mann–Whitney test compared to guides with 0 off-targets. (**b**) Single mismatches closer to the PAM are less tolerated. Guides with no perfect off-targets and exactly one 1-mismatch off-targets were stratified by the location of the mismatch. Those with the location of the mismatch farther from the PAM site display greater disenrichment, demonstrating greater cutting and toxicity at these off-target sites. Error bars represent range of median results from two replicates. (**c**) Median and quartile range of truncated 17 and 18 bp guides in three cell lines by number of 1-mismatch off-targets. Guides with any 0-mismatch off-targets were excluded. *P* values are from a single-tailed Mann–Whitney test compared to guides with 0 off-targets. (**d**,**e**) Box plots of enrichment scores for guides targeting hit genes at 10% FDR for (**d**) growth screens and (**e**) a ricin screen. Signs have been flipped for ricin resistant genes for comparison. Box is length of quartile, whiskers represent 1.5 × quartile, and dots indicate enrichment of outlier guides. *P* values calculated using a single-tailed Mann–Whitney test. NS indicates non-significance (*P*>0.01).

Having observed safe-targeting guide toxicity (Supplementary Fig. 5)—a growth effect independent of a gene effect—we investigated whether we could detect toxicity due to off-target cutting of gene-targeting sgRNAs (Supplementary Data 7). We found that when full-length (19–20 bp) guides have exact off-targets (0-mismatch off-targets) or 1-mismatch off-targets anywhere in the genome, they are more toxic than their counterparts without off-target matches (Fig. 2a; Supplementary Fig. 7). For full-length guides with 2-mismatch off-targets, a significant amount of toxicity is only observed for guides with 5+ off-target sites (Supplementary Fig. 8). Note that all guides were included in this analysis and that excluding guides targeting essential genes does not change these conclusions (Supplementary Fig. 9a,b).

To test the sensitivity of our use of toxicity as a measurement of off-target cutting, we examined the effect of a single mismatch at each nucleotide position. It has been previously reported that the tolerance of a guide to a 1-mismatch off-target depends on where the mismatch lies along the guide, with mismatches closer to the PAM site being less tolerated^32^,^48^,^49^,^50^. As expected, using the ∼10,000 full-length (19–20 bp) gene-targeting sgRNAs with a single 1-mismatch off-target site elsewhere in the genome, we observe that guides with a mismatch more distal from the PAM are more toxic than sgRNAs with a mismatch closer to the PAM (mismatch position versus average median value of two replicates; Pearson rho>0.7; *P* value<0.001; Fig. 2b; Supplementary Fig. 9c). Previous results have also found that high GC content sgRNAs suffer greater off-target activity^32^. Consistent with this, we find that low GC, full-length (19–20 bp) sgRNAs with exactly one 1-mismatch site are significantly less toxic (GC content versus enrichment; Pearson rho<−0.03; *P* value<0.001) than high GC, full-length sgRNAs (Supplementary Fig. 10). Together, these results demonstrate—using ∼10,000 sgRNAs—that toxicity can be used as a sensitive measure of Cas9 cutting and reproduces previously demonstrated features of sgRNAs that influence off-target activity.

### On- and off-target activity of truncated sgRNAs

Since our library contains truncated and full-length sgRNAs, and we can measure off-target cutting using toxicity, we sought to directly compare their relative performance in high-throughput screens. For the ∼10,000 truncated (17–18 bp) guides with a single 1-mismatch off-target site, we observed greatly reduced off-target activity (compare Fig. 2a,c, Supplementary Figs 11a,c,d and 12) and no clear dependence on mismatch position (Supplementary Fig. 11b).

The greater toxicity of full-length guides can still be seen when examining sgRNAs that target essential genes but have no 0- or 1-mismatch off-targets (Fig. 2d; Supplementary Fig. 13a). Notably, when examining genes whose deletion increases the rate of growth, we still observe that full-length guides are more toxic (Supplementary Fig. 13b). This result raises the question of whether truncated sgRNAs may have reduced off-target activity due to reduced overall activity, leading to a trade-off between on- and off-target activities for Cas9 deletion libraries. If truncated guides have major reductions in on-target activity, then truncated sgRNAs targeting ricin regulators would have reduced phenotypes in the screen for ricin regulators in K562. Unlike in growth screens, where cutting at non-genic sites results in measurable toxicity (Supplementary Fig. 5), off-target sites in genes that influence ricin sensitivity should be rare and thus not confound on-target activity (Supplementary Fig. 6a,b). We found a minor but not significant (*P*>0.01) increase in activity with longer guides as indicated by slightly greater depletion for ricin sensitizers or greater enrichment for protective hits (Fig. 2e; Supplementary Fig. 13c). Thus, our data do not necessarily indicate that truncated sgRNAs have equivalent cutting efficiency, only that they appear effective in high-throughput screens. These results support the conclusion that for screening applications, truncated guides provide fewer off-target effects with no major reduction in on-target efficacy.

## Discussion

Here we developed a new genome-wide CRISPR-Cas9 based library with variable-length sgRNAs and safe-targeting controls and used it to examine how Cas9 toxicity and off-target cutting can affect genome-wide Cas9 deletion screens in three cell lines. Using toxicity as a sensitive measure of Cas9 off-target activity, we were able to measure cutting only at 0-mismatch, 1-mismatch, and 2-mismatch off-target sites. While Cas9 nuclease has been shown to tolerate many more mismatches, these cutting events may occur at too low a frequency to significantly influence high-throughput screens. To correct for the effects of nuclease toxicity we developed safe-targeting sgRNAs—directed towards sites with minimal predicted functional impact—as more appropriate negative controls in CRISPR-Cas9 experiments. Finally, we have demonstrated with thousands of guides the reduced off-target activity of truncated sgRNAs without major loss of on-target efficacy in the context of high-throughput screens.

While the presented library was designed to test hypotheses about sgRNA length, controls, and off-targets, it also represents a robust tool for genome-wide CRISPR-Cas9 deletions screens. Existing gold-standard sets for essential genes^38^ allow the direct measurement of the library's high performance across multiple cell lines (Fig. 1c). While no such gold-standard set exists for ricin regulators, the identification of previous hits and the completeness of known pathways controlling ricin susceptibility provides strong evidence for high performance of this library in selection screens as well (Fig. 1d).

We present a class of safe-targeting guides to control for the DNA damage caused by gene-targeting guides. In theory, these should better recapitulate the non-specific effects of gene-targeting guides, and in fact we demonstrate that they behave significantly differently from non-targeting controls in both growth and non-growth screens (Supplementary Figs 5 and 6a,b). As this has a real effect on the screen results (Fig. 1e; Supplementary Fig. 6c), these safe-targeting guides may provide a more appropriate negative control than widely used non-targeting guides. Note this is similar in principle to the use of sgRNAs targeting gold-standard nonessential genes as negative controls to recapitulate the effects of cutting^38^,^51^. Interestingly, safe-targeting guides do not behave identically to sgRNAs targeting gold-standard nonessential genes in growth screens (Supplementary Fig. 5), which may be due to distinct cutting behaviour or the presence of weakly essential genes in the gold-standard nonessential set. While we demonstrate the use of safe-targeting guides in the context of high-throughput growth (Fig. 1e) and non-growth screens (Supplementary Fig. 6c), they likely represent more appropriate controls for low-throughput experiments as well.

Using the measurable growth phenotype caused by Cas9 nuclease activity, we developed a method to profile off-targets in high throughput (Fig. 2a). We recovered known effects of GC content and mismatch position on off-target cutting (Fig. 2b; Supplementary Fig. 10) and detect off-targets at sites up to 2-mismatches (Supplementary Fig. 8). These conclusions hold true across multiple cell lines, though the effect is reduced for U937 (Fig. 2a,b,d); indeed, off-target effects may differ depending on Cas9 expression level, genetic background or other differences between cell lines. We note that these results appear to contrast with highly sensitive genome-wide off-target profiling methods such as GUIDE-seq^32^, Digenome-seq^27^,^28^, and BLESS^26^, which monitor DNA breaks and have observed significant cutting at off-target sites with up to 6 mismatches^27^,^28^,^32^. We are not able to measure growth effects from cutting at such sites, suggesting that the vast majority of these cutting events may occur at too low of a frequency to have a detectable effect on cell fitness in our assay. The key advantage of our use of growth phenotype as a proxy measurement for off-target cutting is that it allows us to assay tens of thousands of guides in a single experiment. Though our assay cannot directly measure cutting efficiencies of sgRNAs or detect individual off-target events, by measuring off-targets across thousands of guides, we can extract generalizable conclusions about off-target sites and evaluate strategies to reduce off-target cutting in high-throughput screens. While we measure the effect of off-targets in growth screens, these conclusions should be relevant to preventing off-target effects in non-growth screens as well.

These results establish a convenient and robust method for detection of on- and off-target efficacy of sgRNAs and Cas9 variants in high-throughput, demonstrate a new strategy to use safe-targeting controls to more accurately perform hit selection in Cas9 screens, and may help define new rules for the design of sensitive and specific Cas9 knockout libraries.

## Methods

### Gene-targeting guides

Exonic guide sites fitting the pattern G(N_16–19_)NGG were selected. For cases where multiple lengths are possible, only the longest guide was used. These guide sites were then annotated as targeting Ensembl GRCh37 genes models to generate candidate guides towards each gene. For each candidate guide, the following features were annotated: The coding percentage from the 5′ end, the fraction of transcript models the targeted exon appeared in, and the exon number. For genes with multiple transcript models, the median metric across each model was taken. Additionally, the number of off-target sites in the genome up to 4 mismatches was calculated, considering only G(N_16–19_)NGG as possible off-targets with a two-basepair seed region.

Candidate guides were removed if they contained restriction enzyme sites necessary for cloning or TTTT homopolymers, which indicate transcription stop from the U6 promoter driving sgRNA expression, as well as those with GGGGG adjacent to the PAM which prevents sequencing on a NextSeq using our sequencing strategy. Guides were then ranked on a weighted scheme for features expected to influence on- and off-target activity. Guides were given 1,000 points for each 0-mismatch off-target, 100 points for each 1-mismatch off-target, 10 points for each 2-mismatch off-target, 500 points times the percentage through the coding region from the translational start, 500 points times the percentage of coding models the targeting site was not included in and 1,000 points if the GC content of the guide was <20% or >80%. To ensure the library would be equally split between full-length (19–20 bp) and truncated (17–18 bp) sgRNAs, full-length guides were given an extra 100 points. Guides were than ranked from lowest to highest number of points. For example, if a guide had no 0-mismatch off-targets, two 1-mismatch off-targets and 10 2-mismatch off-targets, then it would receive 0 × 1,000+2 × 100+10 × 10=300 points. If the guide was located in an exon present in four fifths of transcript models, then it would receive 500(1−4/5)=100 points. If the targeting site was 20% through the coding region from the translational start, it would receive 500 × 0.2=100 points. If the guide had normal GC content and was truncated, it would receive no additional points for a total of 300+100+100=500 points. It would then be ranked against all other guides targeting that gene, from lowest to highest points.

After this initial ranking, additional penalties were used to select more variable guides: If a guide overlapped a higher ranking guide, 500 points was given. If a guide targeted the same exon as five higher ranking guides, 500 points was given. These additional penalties were given based only on the original ranking. The top 10 guides towards each gene, those with the lowest score, were then selected (Supplementary Data 1 and 2). Note that for genes with few candidate guides, this results in the inclusion of poor quality guides. Relative penalties were selected based on the observed distribution of guide qualities.

### Non-targeting guides

To design non-targeting negative control guides with similar properties to the targeting guides, the selected gene-targeting guides were scrambled and tested for intended properties. Each targeting guide was used to generate a candidate non-targeting guide sequence by retaining the nucleotide composition and length of the guide and permuting the sequence. Candidate non-targeting guides were not considered if they contained 5′-GGGGG-3′ or 5′-TTTT-3′ homopolymers or restriction sites. To ensure that non-targeting guides had no targets in the genome, the 17 PAM-proximal nucleotides were mapped to the genome with BWA^52^ using both the NAG and NGG PAMs, and sequences which mapped with zero or one mismatch was permuted and tested again. Guides were repeatedly tested in this manner until a guide towards at least 95% of targeted genes had an acceptable permuted version. Of these, 10,000 guides were selected randomly to form the complete set of non-targeting guides, and 5,644 of these were chosen randomly to be included in the library (Supplementary Data 5).

### Safe-targeting guides

We defined safe regions as genomic regions without detectable signals across a range of biochemical assays and sequence-based analyses. We performed this analysis on the human hg19 assembly. We first identified the regions classified in inactive chromatin states (Quies, ReprPC. ReprPCWk or Het) across all available cell types in the Roadmap Epigenomics project^53^. The intersection of these gives the genomic regions that are inactive in every one of these cell types. From these, we filtered out the following: conserved elements, as defined from GERP34, phastCons32PlacentalMammals, phastCons46Vertebrates, phastCons9Primates, SiPhy29Mammals, DNase peaks from the ENCODE project^54^, repeats downloaded from IGV browser tracks (SINE, LINE, LTR, DNA, Simple_repeat, Low_complexity, Satellite, RC, RNA, Other Unknown), transcription factor binding motifs defined in the ENCODE project across the hg19 genome to find significant motif matches^55^, transcription factor binding sites as defined by ChIP-seq experiments from the ENCODE projects using the irreproducible discovery rate (IDR) pipeline^56^, sites on the genome blacklist^54^, unmappable regions, gene exons and UTRs from GENCODE v19 (ref. 57), and transcription start sites from combined analysis of Gencode annotations and CAGE-seq data from the Fantom5 consortium. Given that some of the criteria are not available on chrX, there is an enrichment of safe regions for that chromosome. Thus, we selected 10,000 safe-targeting controls evenly distributed across chromosomes and included 6,750 of these based on off-targets and GC content (Supplementary Data 5).

### Cell culture

Cell culture performed as previously described^35^. Briefly, K562 (ATCC) and Ramos (ATCC) were cultured in RPMI 1640 (Gibco) media and supplemented with 10% FBS (Hyclone), penicillin (10,000 I.U ml^−1^), and L-glutamine (2 mM). U937 (ATCC) were cultured RPMI 1640 (Gibco) media and supplemented with 10% heat-inactivated FBS (Hyclone) and penicillin (10,000 I.U ml^−1^). Cells were grown in log phase during all biological assays by returning the population to 500,000 cells per ml each day. K562, Ramos, and U937 cells were maintained in a controlled humidified incubator at 37 °C with 5% CO_2_.

### Screening

Pooled, genome-wide CRISPR deletion screens were performed in three cell lines: K562 stably expressing SFFV-Cas9-BFP, Ramos cells lentivirally infected with SFFV-Cas9-BFP, and U937 cells lentivirally infected with EF1a-Cas9-Blast^34^. The library was synthesized, cloned and lentivirally infected into cells as previously described^20^. Briefly, the parent vector for the libraries was derived from a pSico lentiviral vector which expresses GFP and a puromycin-resistance cassette separated by a T2A sequence^45^,^58^; we replaced GFP with mCherry to make the final parent vector, pMCB320. Sublibraries were PCR-amplified from pooled-oligo chips (CustomArray, Agilent), digested with BstXI and BlpI restriction enzymes, and ligated into BstXI/BlpI-cut pMCB320 using T4 ligase. Libraries and vectors will be made available via Addgene. Three days after infection, cells were placed under puromycin selection (0.7 μg ml^−1^, Sigma) for an additional 3 days after infection, then split at time 0. Throughout the screen, the pooled libraries were maintained at 1,000 cells per guide or a total of ∼250 million cells in large spinner flasks. K562 and U937 were grown for ∼2 weeks, and Ramos cells were growth for ∼3 weeks due to their slower division time. Genomic DNA was extracted following Qiagen's Blood Maxi Kit, and the guide composition was sequenced and compared to the plasmid library using casTLE^20^ version 1.0 available at https://bitbucket.org/dmorgens/castle. Briefly, casTLE compares each set of gene-targeting guides to the negative controls, selecting the most likely maximum effect size which explains the distribution of targeting guides. It then determines the significance of this maximum effect by permuting the results^20^. Both safe-targeting and non-targeting controls were used for this analysis. For the ricin sensitivity screen, cells were treated with ricin toxin (Vector Labs) at 0.25 ng ml^−1^ for 24 h, ricin was removed and then cells were allowed to recover to normal doubling rate. This treatment occurred four times over 2 weeks.

### Off-target analysis

Genome-wide off-target sites with up to 2 single-nucleotide mismatches were found via the BWA alignment software^52^ with no seed region (Supplementary Data 7). Enrichment values for each guide in each screen were calculated as a log ratio of counts, normalized for sequencing depth and the median enrichment of both non-targeting and safe-targeting negative controls as previously described^20^.

### Data availability

All sequencing data used for the screens is available from the authors. Count files containing element-wise summaries of the sequencing data are available as Supplementary Data 3. Full gene-wise summaries of screens are also available as Supplementary Data 4. Off-target data used for all figures is available as Supplementary Data 7.

## Additional information

**How to cite this article:** Morgens, D. W. *et al*. Genome-scale measurement of off-target activity using Cas9 toxicity in high-throughput screens. *Nat. Commun.* **8,** 15178 doi: 10.1038/ncomms15178 (2017).

**Publisher's note**: Springer Nature remains neutral with regard to jurisdictional claims in published maps and institutional affiliations.

## Supplementary Material

Supplementary Information Supplementary figures.

Supplementary Data 1 Human CRISPR-Cas9 library. Guide sequences and sublibrary breakdown for the 10-guide human library.

Supplementary Data 2 Mouse CRISPR-Cas9 library. Guide sequences and sublibrary breakdown for the 10-guide mouse library.

Supplementary Data 3 Count files for all screens. Files containing counts for each guide sequence in each condition.

Supplementary Data 4 Screen results. Full casTLE results for each screen.

Supplementary Data 5 Human and mouse controls. Lists of all non-targeting guides. Lists of all designed safe-targeting guides, including those not used in screens. Lists of safe regions used to select guides.

Supplementary Data 6 Non-targeting vs safe-targeting. Lists of genes that are differentially identified when using either non-targeting or safe-targeting controls.

Supplementary Data 7 Summary of off-target information. Number of predicted off-targets for each guide in each screen.
