# Supplementary material for: Genome-scale measurement of off-target activity using Cas9 toxicity in high-throughput screens
Source: Nat Commun. 2017 May 5;8:15178. doi: 10.1038/ncomms15178 (PMC5424143; doi:10.1038/ncomms15178)
Supplement: Supplementary Information — Supplementary figures. [file ncomms15178-s1.pdf]

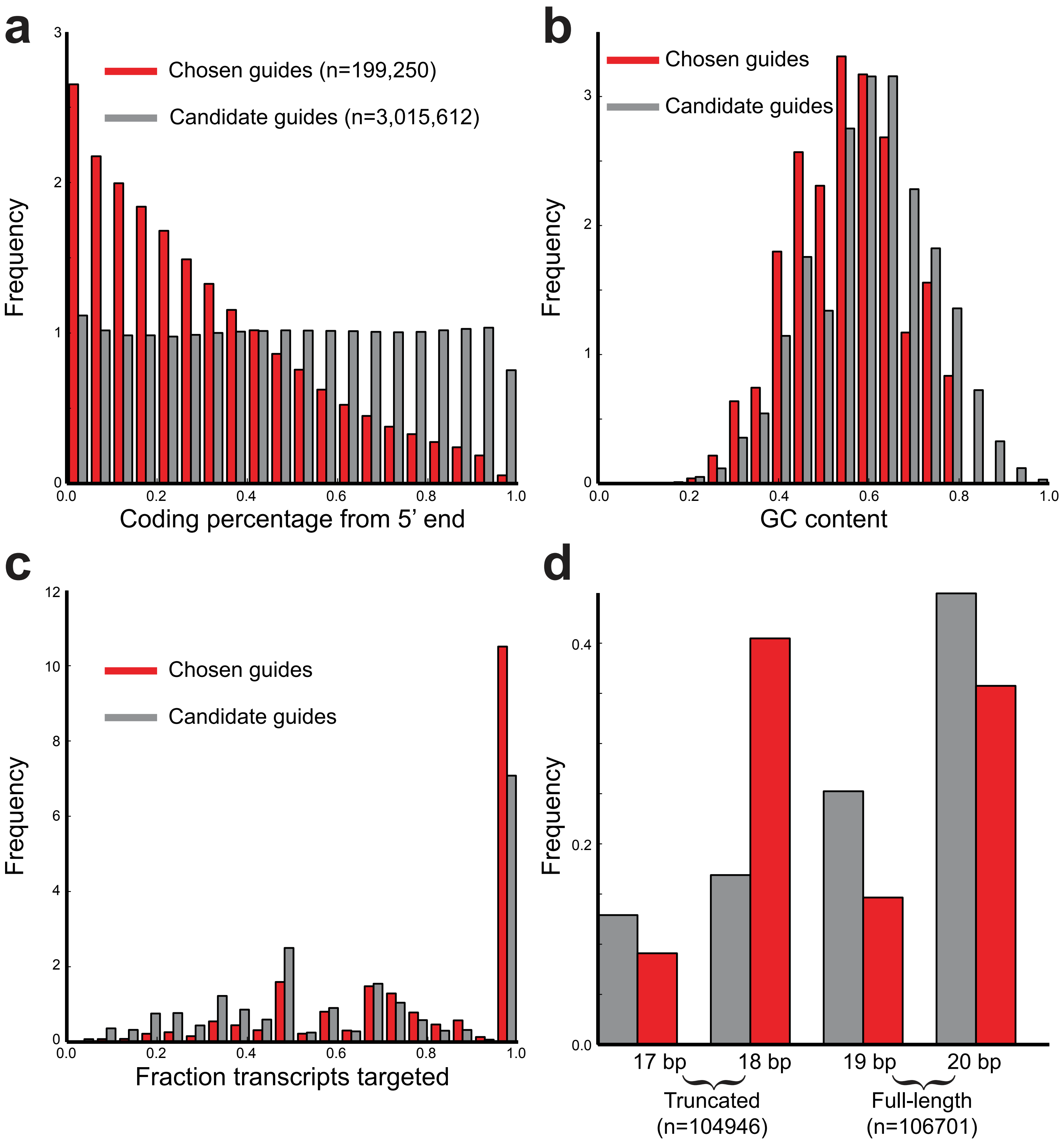

Supplementary Figure 1: Features of guides included in library. a) Candidate and chosen distribution of guide sites along gene length. Guides sites closer to the 5' end were favored. b) Candidate and chosen distribution of GC content of guides. Guide sites with moderate GC content were favored. c) Candidate and chosen distribution of conservation of target site among transcript models. Guide sites present in a greater fraction of transcripts were favored. d) Candidate and chosen distribution of guide lengths. Truncated guides were favored in order to select an even number of truncated and full length guides.

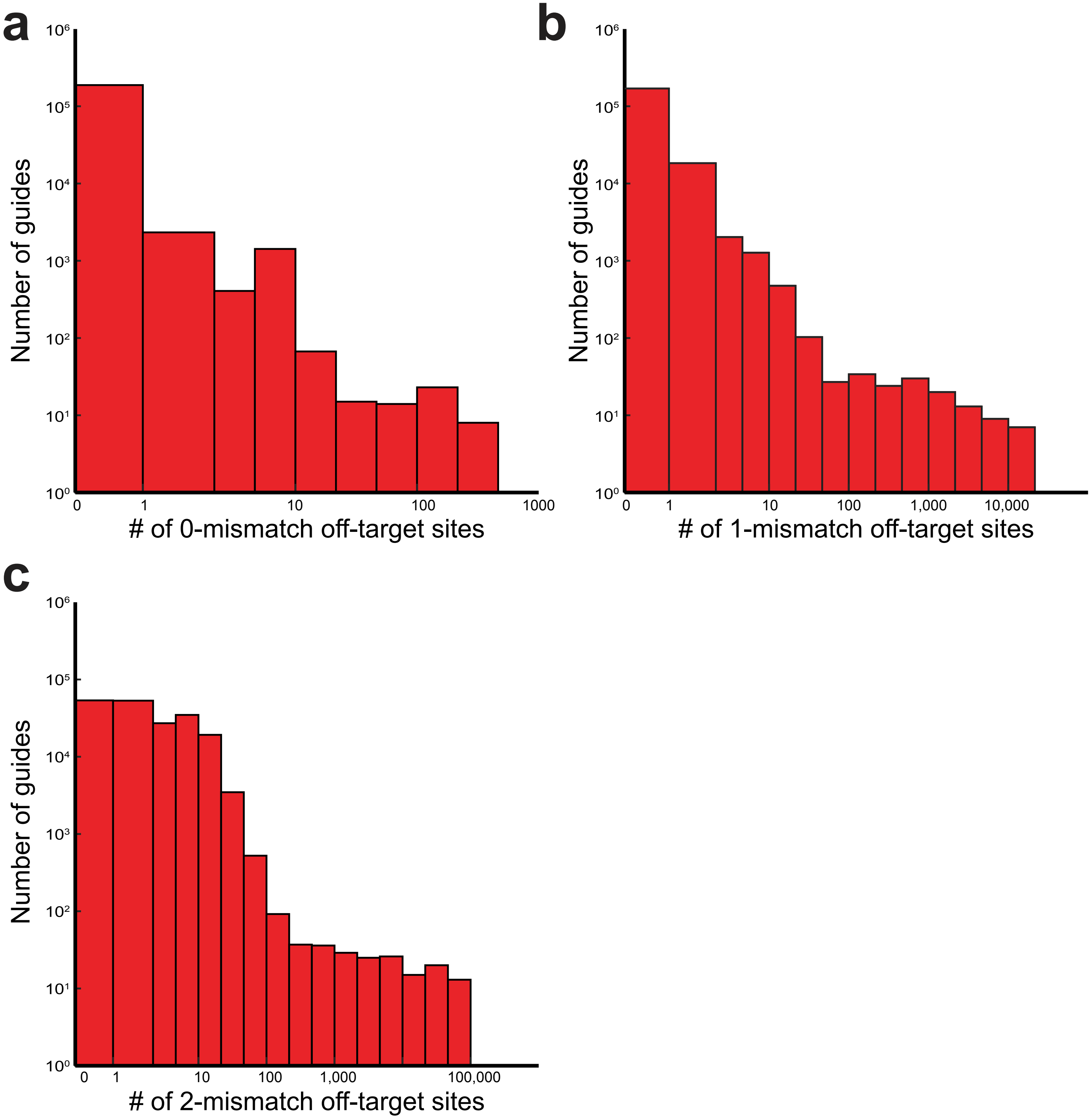

Supplementary Figure 2. Distribution of off-target sites in the library. Histogram represents the number of guides in the library with certain numbers of off-targets, as defined by 0, 1, and 2-mismatch sites. a-c) Number of off-targets in library for a) 0-mismatch sites, b) 1-mismatch sites, and c) 2-mismatch sites.

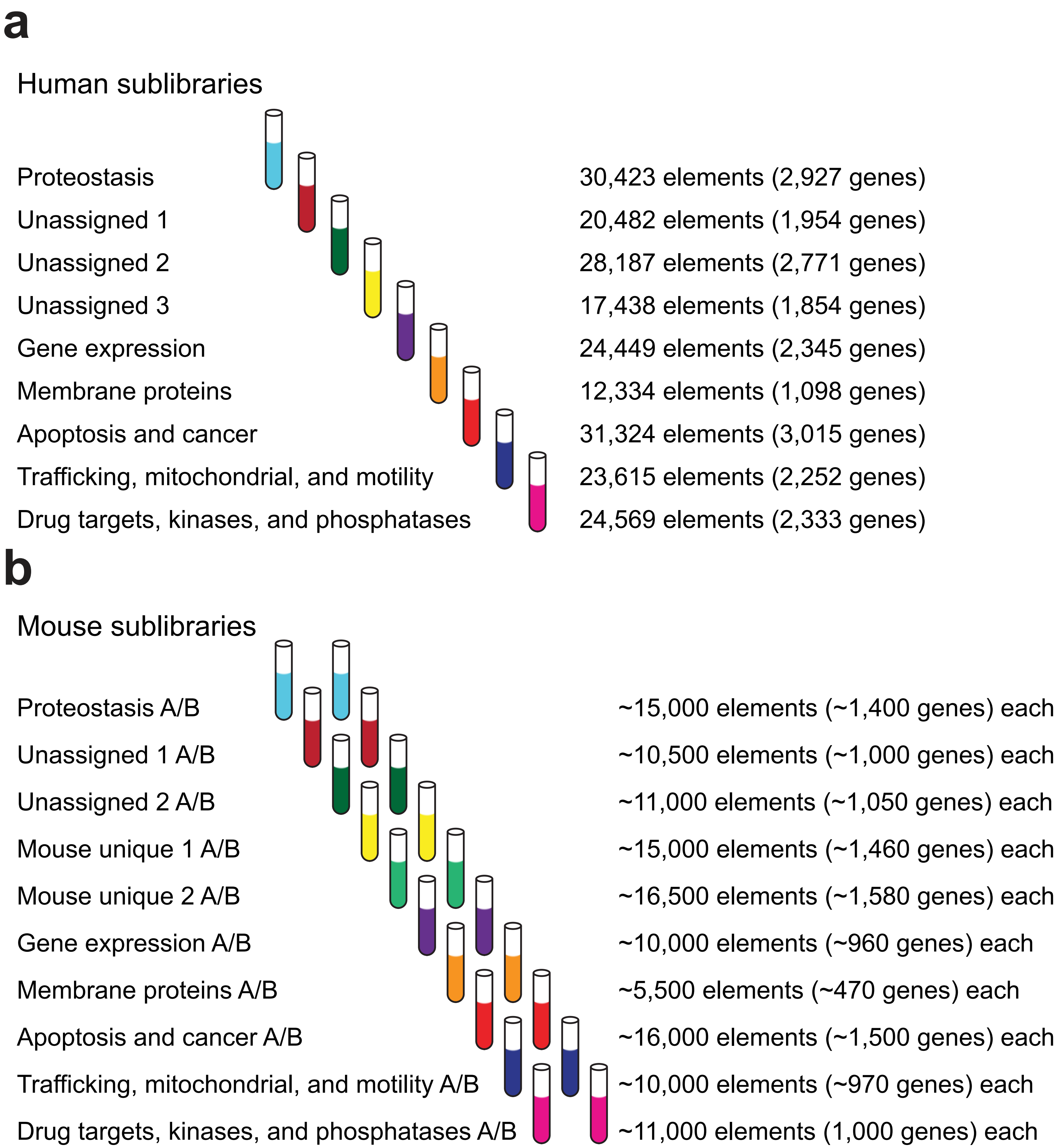

Supplementary Figure 3. Sublibrary composition of libraries. a) Human library is split into 9 sublibraries defined by biological categories of genes. All ~20,500 protein-coding genes are targeted. b) Mouse library is split into 10 sublibraries defined by biological categories of genes. Each of these is then split randomly by gene into two sublibraries. All ~23,000 protein-coding mouse genes are targeted.

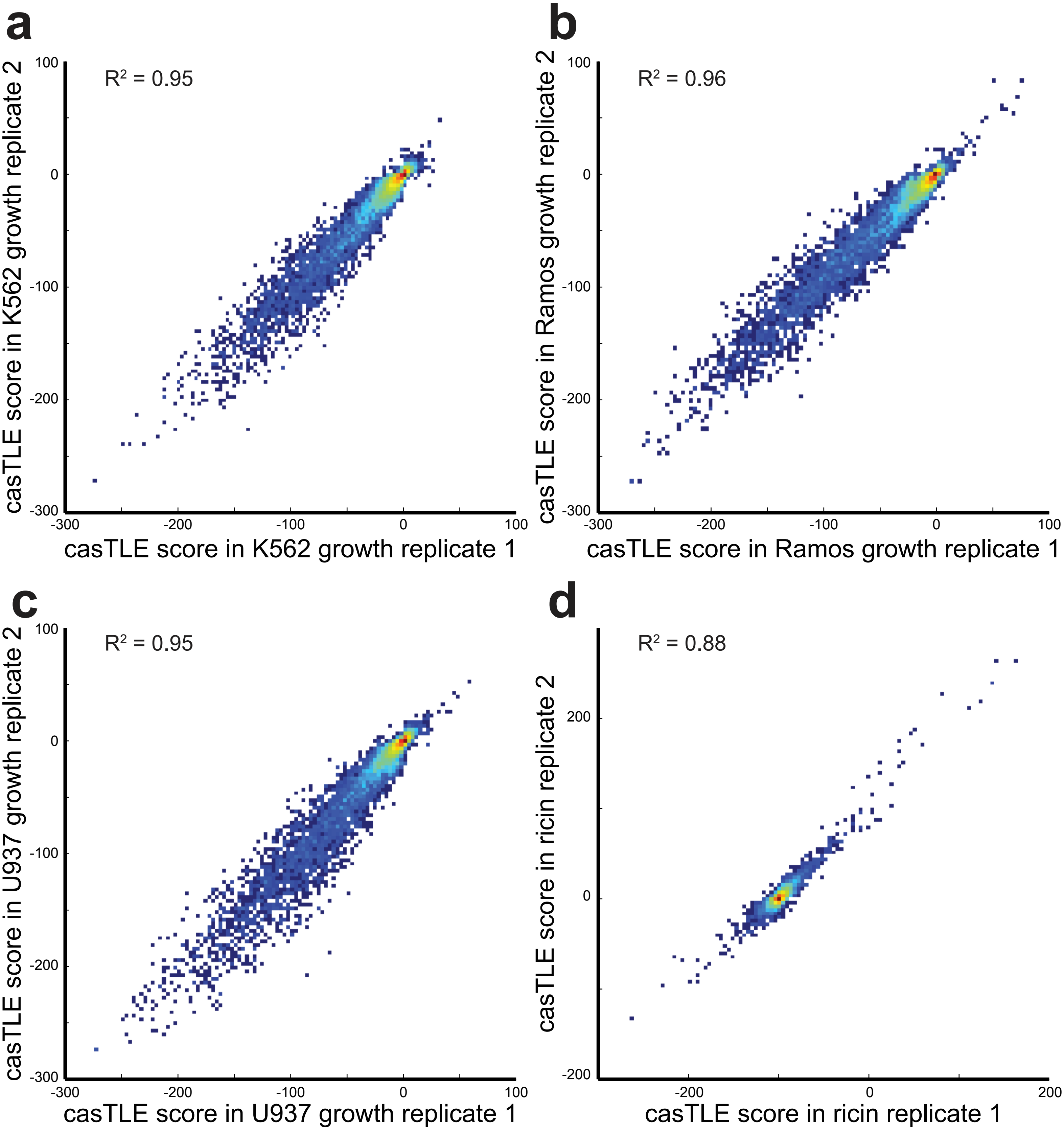

Supplementary Figure 4. Reproducibility of screens. a-c) Reproducibility of casTLE scores for growth screens in a) K562, b) Ramos, and c) U937. d) Reproducibility of casTLE scores in a screen for genetic modifiers of ricin sensitivity in K562. A large positive casTLE score indicates that the gene knockout a-c) increases the growth rate or d) protects the cell from ricin toxicity. A highly negative casTLE score indicates that the gene knockout a-c) reduces the growth rate or d) sensitizes the cell to ricin toxicity.

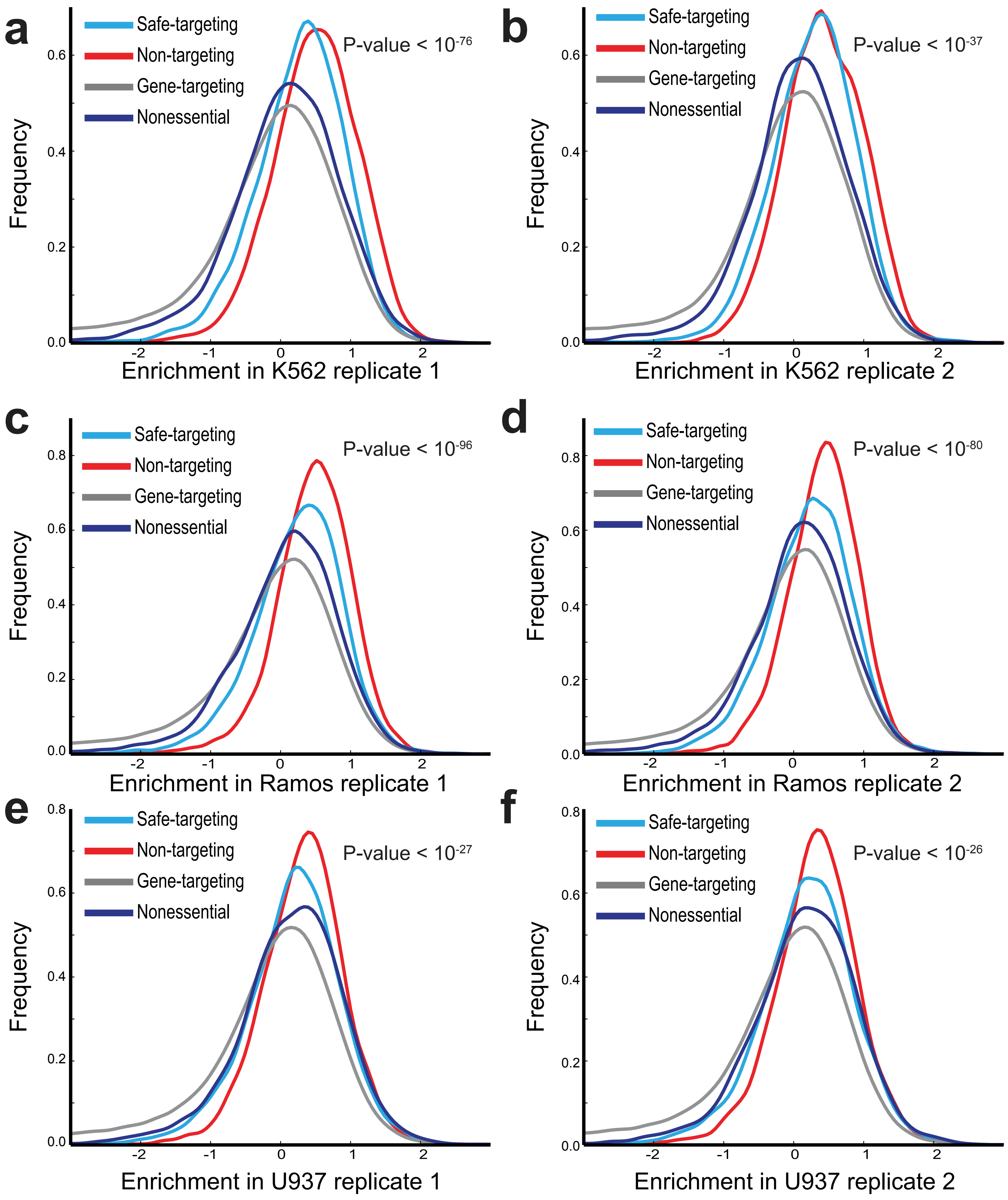
a
Safe-targeting
Non-targeting
Gene-targeting
Nonessential
P-value < 10^-76
Frequency
Enrichment in K562 replicate 1
b
P-value < 10^-37
Enrichment in K562 replicate 2
c
P-value < 10^-96
Enrichment in Ramos replicate 1
d
P-value < 10^-80
Enrichment in Ramos replicate 2
e
P-value < 10^-27
Enrichment in U937 replicate 1
f
P-value < 10^-26
Enrichment in U937 replicate 2

Supplementary Figure 5. Distributions of different classes of guides in growth screens for a-b) K562, c-d) Ramos, and e-f) U937 cells. Safe-targeting refers to guides designed to target regions in the genome with no annotated function. Non-targeting refers to guides designed to have no targets in the genome. Gene-targeting refers to all guides targeting genes in the library. Nonessential refers to guides targeting gold standard nonessential genes[34]. Distributions are calculated as kernel estimations. P-values are calculated using a two-tailed Mann-Whitney test comparing safe-targeting guides to non-targeting guides.

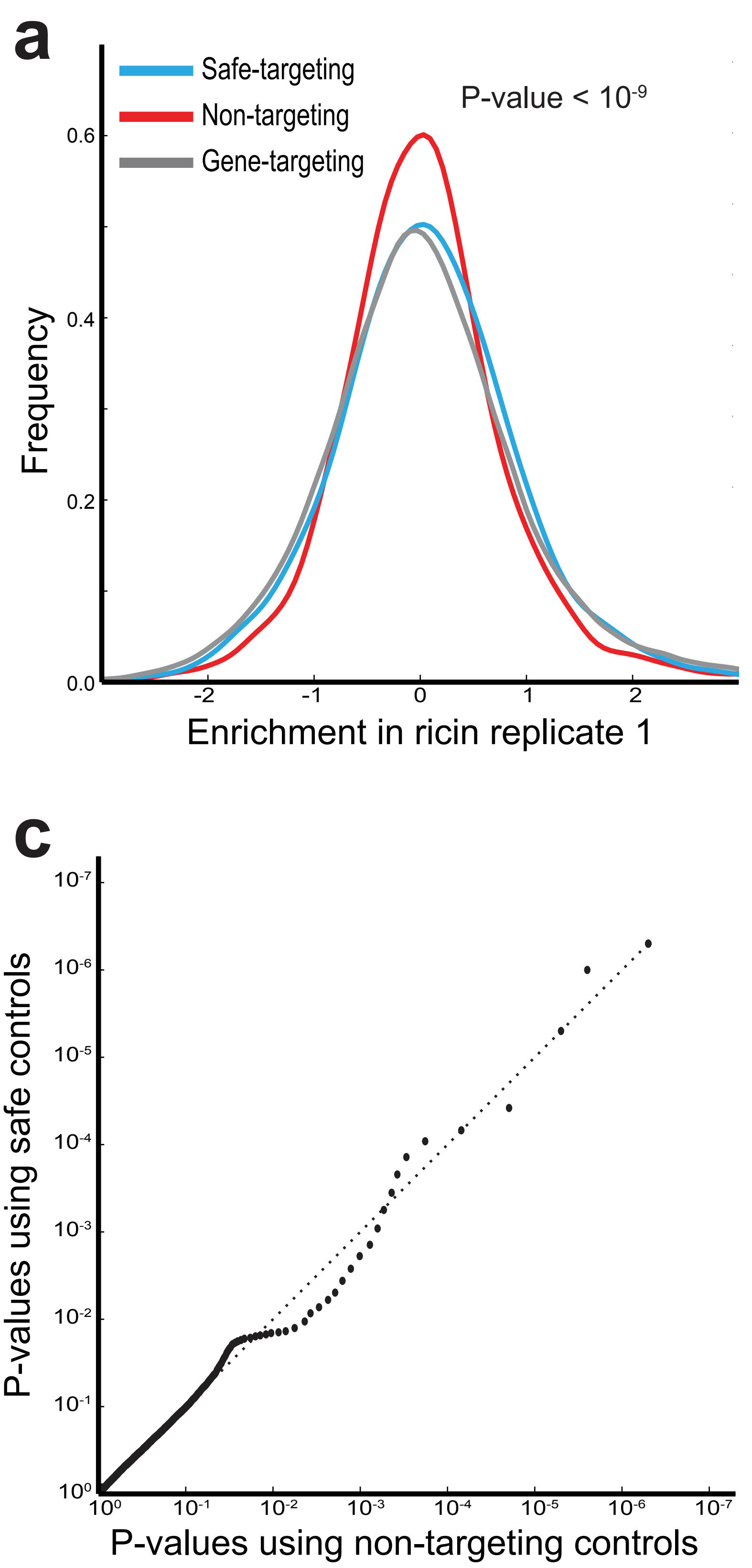

Supplementary Figure 6. Effect of safe-targeting controls on screens. a-b) Distribution of safe-targeting, non-targeting, and gene-targeting guides in a) replicate 1 and b) replicate 2 of ricin screen in K562. P-values are calculated using the Kolmogorov-Smirnov test comparing the distributions of safe-targeting guides to non-targeting guides. c) QQ plot showing altered distribution of P-values using safe-targeting or non-targeting control in a ricin screen. P-values are calculated from both replicates using casTLE[20].

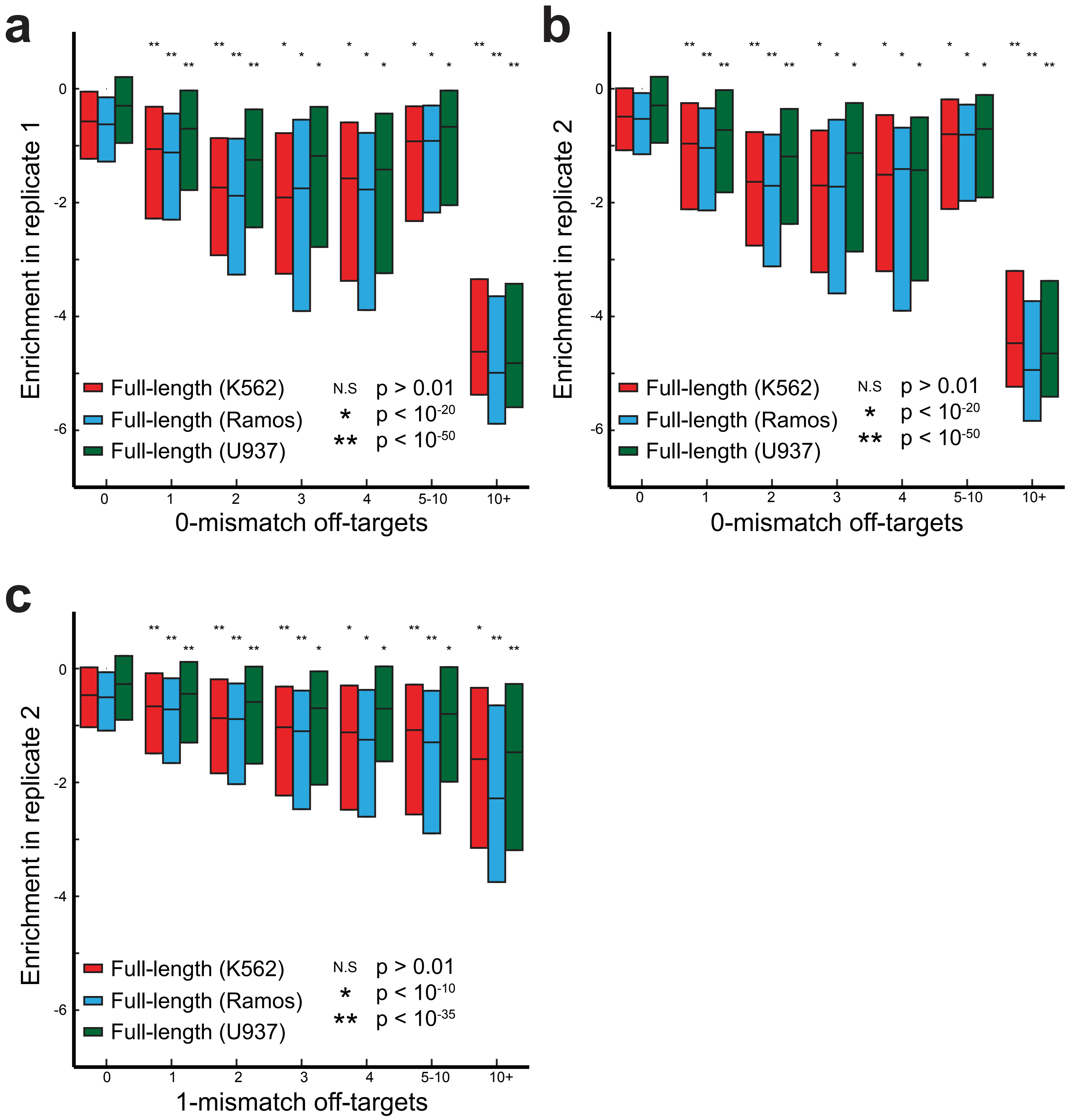

Supplementary Figure 7. Medians and quartile ranges of full-length guides for growth screens by number of off-targets. a-b) Toxicity of perfect matches to the genome for a) replicate 1 and b) replicate 2. c) Toxicity of 1-mismatch off-targets for replicate 2 of growth screens. See Figure 2a for replicate 1. P-values are from a single-tailed Mann-Whitney test compared to guides with 0 off-targets.

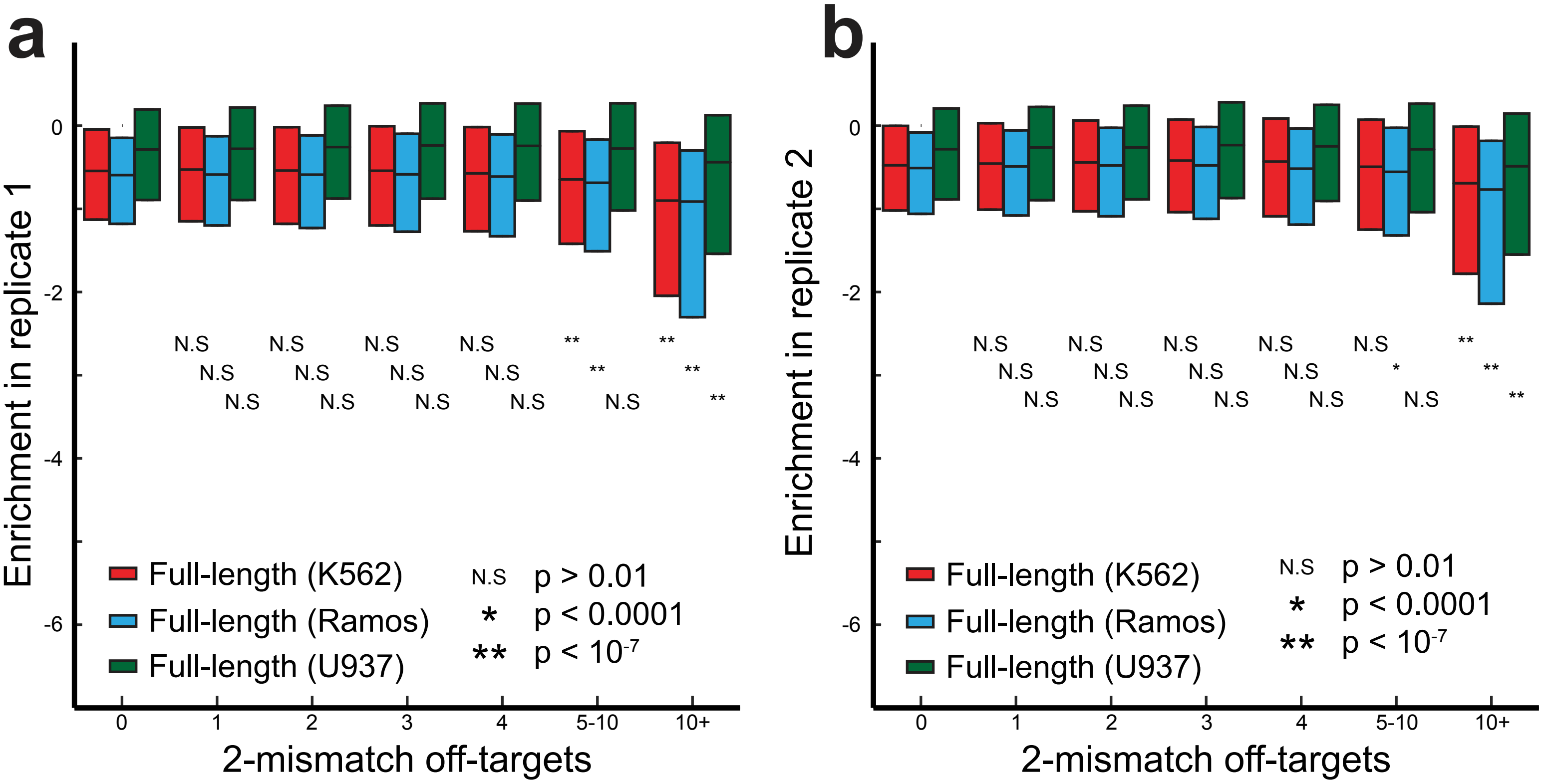

Supplementary Figure 8. Medians and quartile ranges for growth screens by number of 2-mismatch off-targets. a-b) Toxicity of 2-mismatch off-targets in the genome for a) replicate 1 and b) replicate 2. P-values are from a single-tailed Mann-Whitney test compared to guides with 0 off-targets.

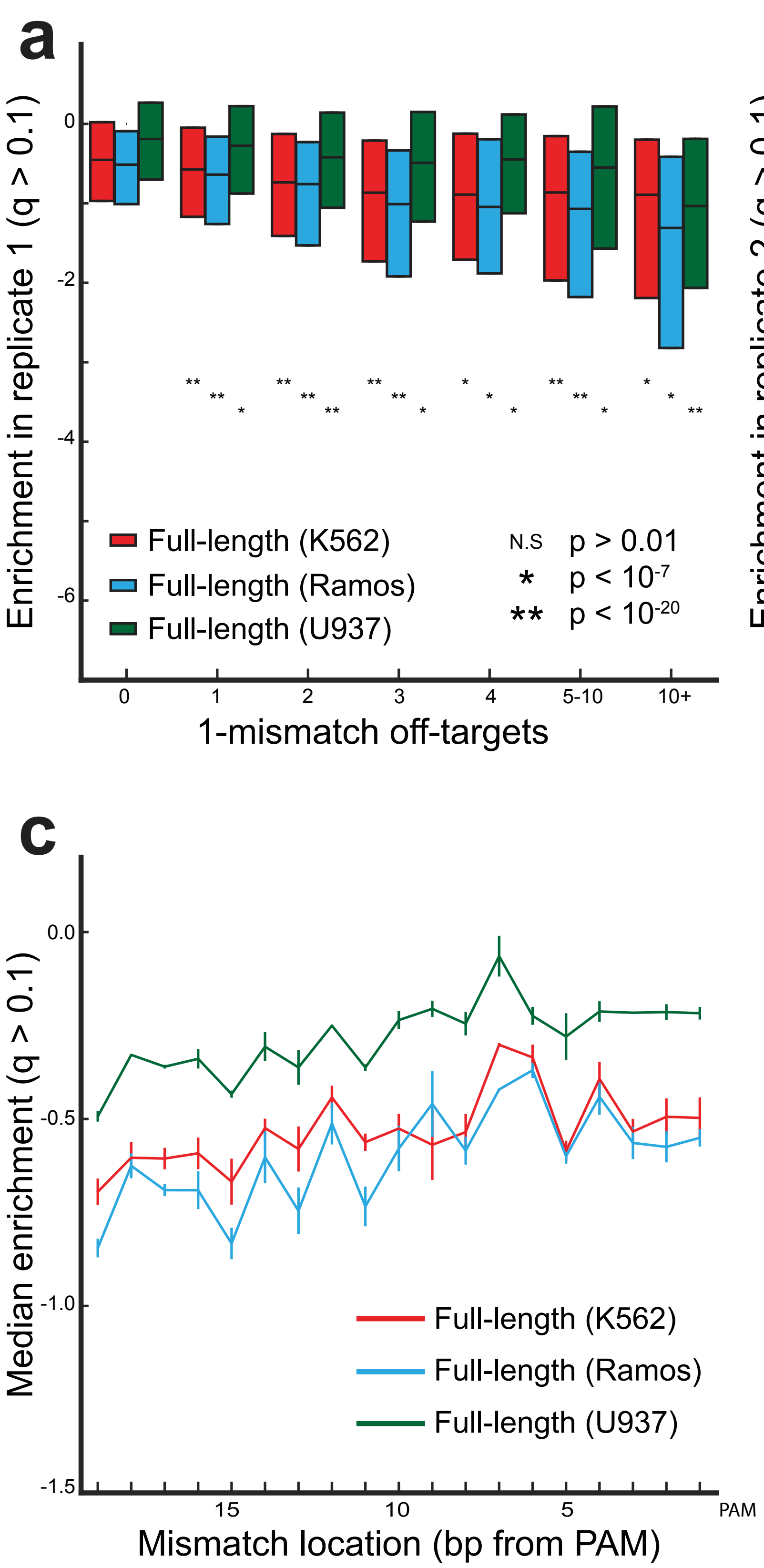

Supplementary Figure 9. Full length (19-20 bp) results after excluding guides targeting essential genes. a-b) Medians and quartile ranges of full-length sgRNAs for growth screens by number of 1-mismatch off-targets for a) replicate 1 and b) replicate 2 after guides targeting essential genes are excluded. P-values are from a Mann-Whitney test compared to guides with 0 off-targets. c) Positional dependence of full-length sgRNAs after excluding guides targeting essential genes. Essential genes determined by combination analysis of two replicates at 10% FDR. P-values are from a single-tailed Mann-Whitney test compared to guides with 0 off-targets.

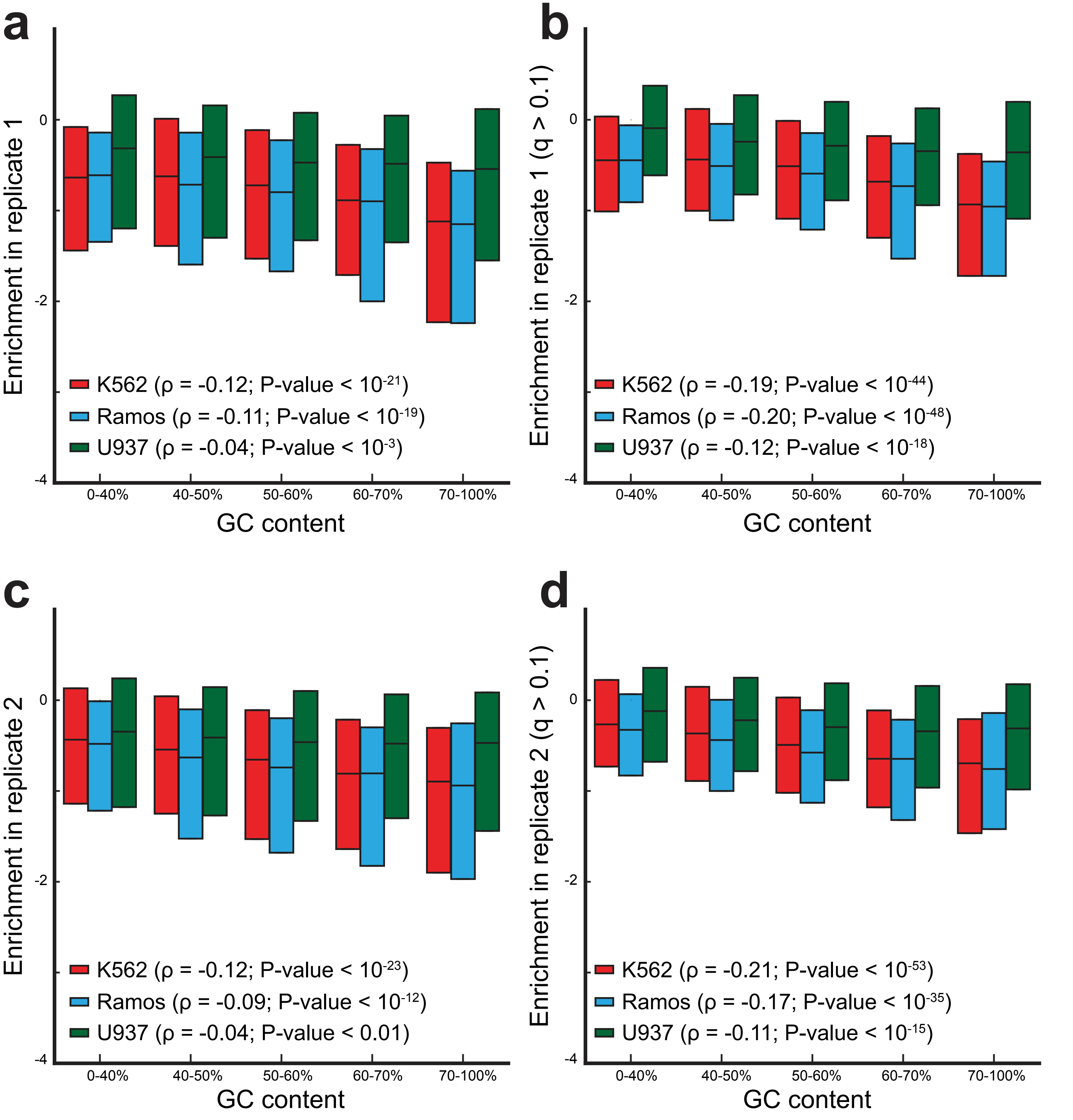

Supplementary Figure 10. Medians and quartile ranges for growth screens by GC content. a,c) Results from all full-length guides with exactly one 1-mismatch site and b,d) results after guides targeting essential genes (chosen at 10% FDR) are excluded. P-values are from Pearson correlation.

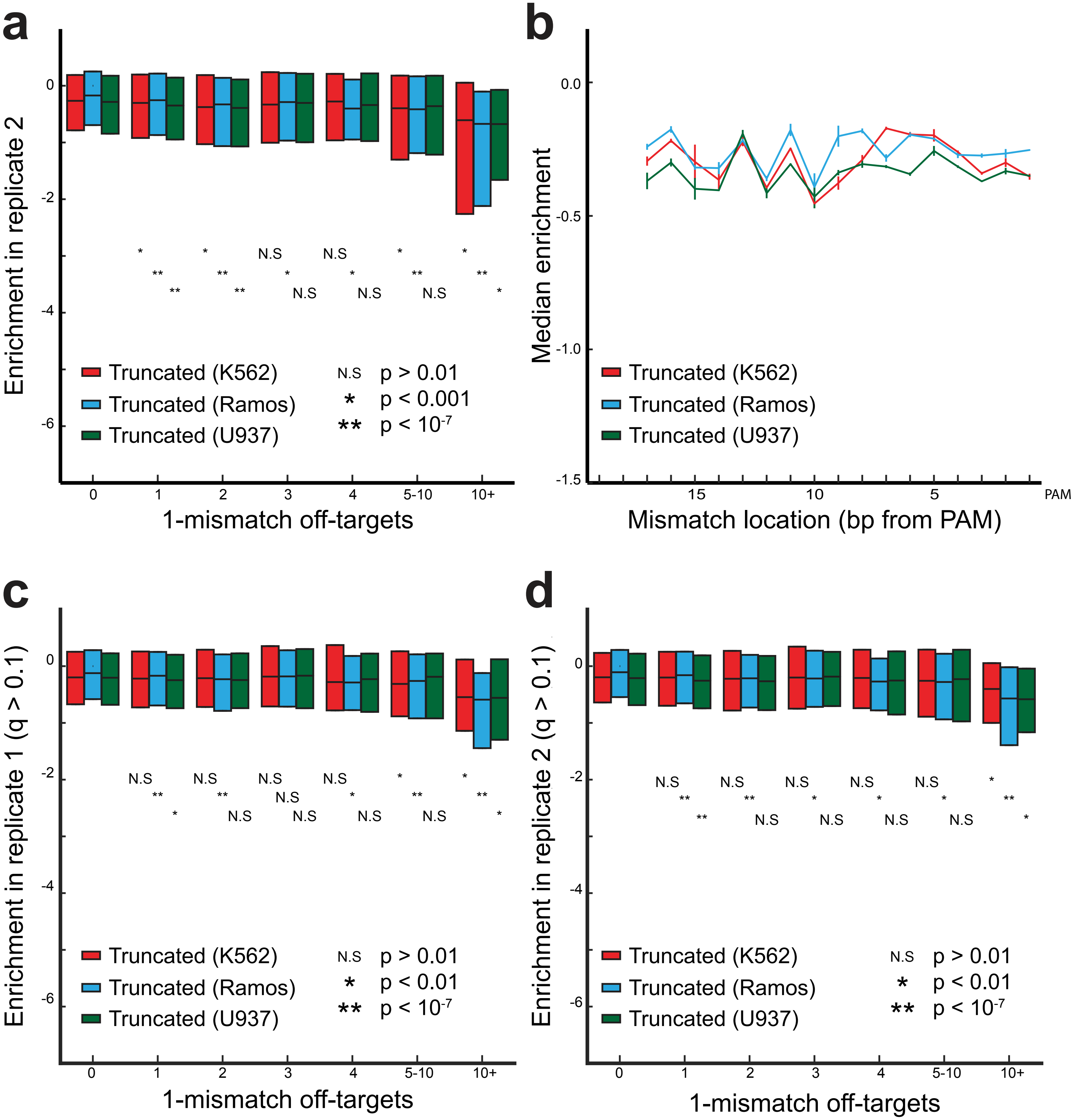

Supplementary Figure 11. Off-target results for truncated (17-18 bp) guides. a) Medians and quartile ranges of truncated sgRNAs for growth screens by number of 1-mismatch off-targets. b) Positional dependence of truncated sgRNAs. c-d) Medians and quartile ranges of truncated sgRNAs for growth screens by number of 1-mismatch off-targets after excluded genes targeting essential genes at 10% FDR. P-values are from a single-tailed Mann-Whitney test compared to guides with 0 off-targets.

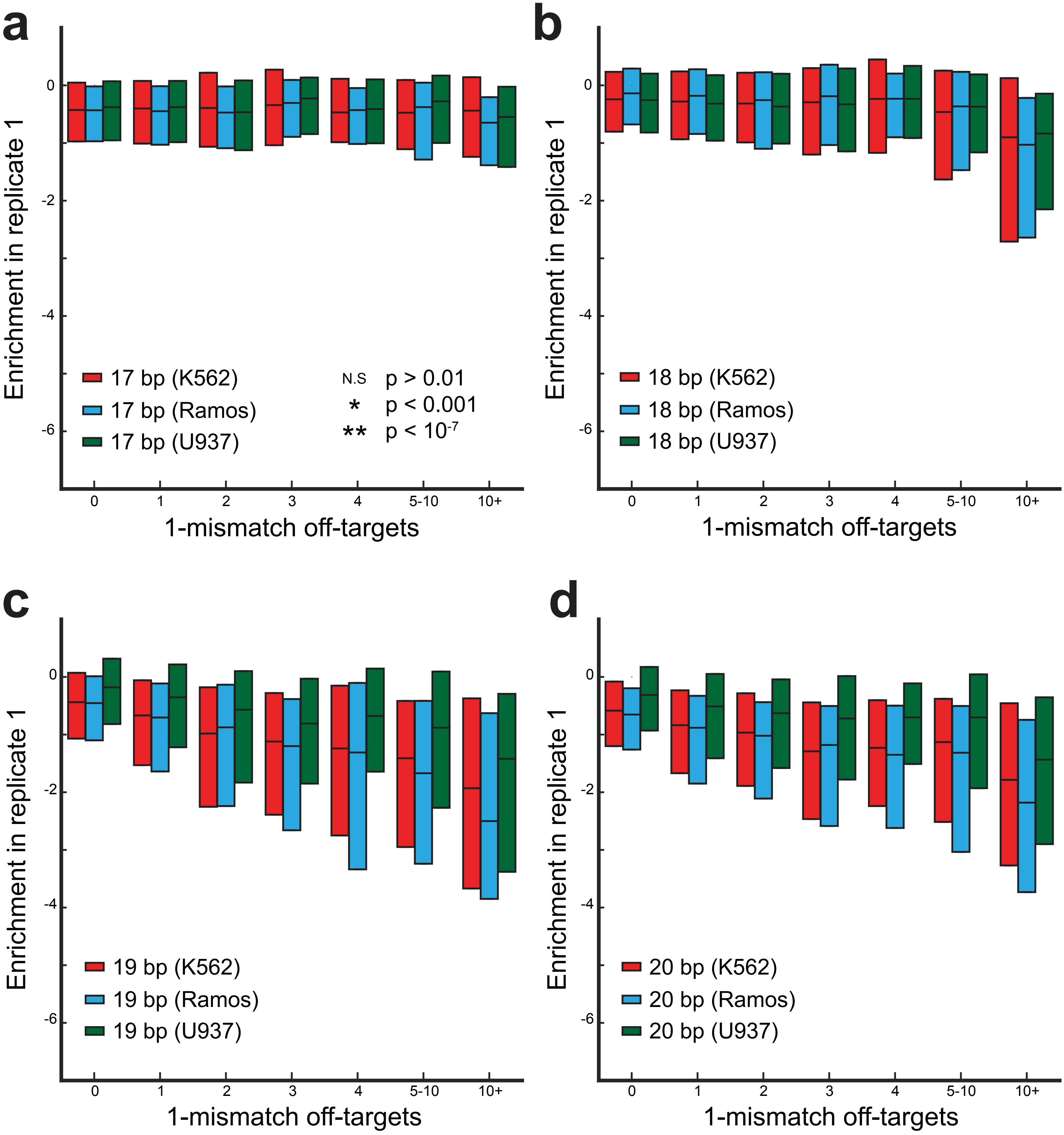

Supplementary Figure 12. Off-target results by length. Medians and quartile ranges of sgRNAs for growth screens by number of 1-mismatch off-targets for guides of length a) 17 bp, b) 18 bp, c) 19 bp, and d) 20 bp.

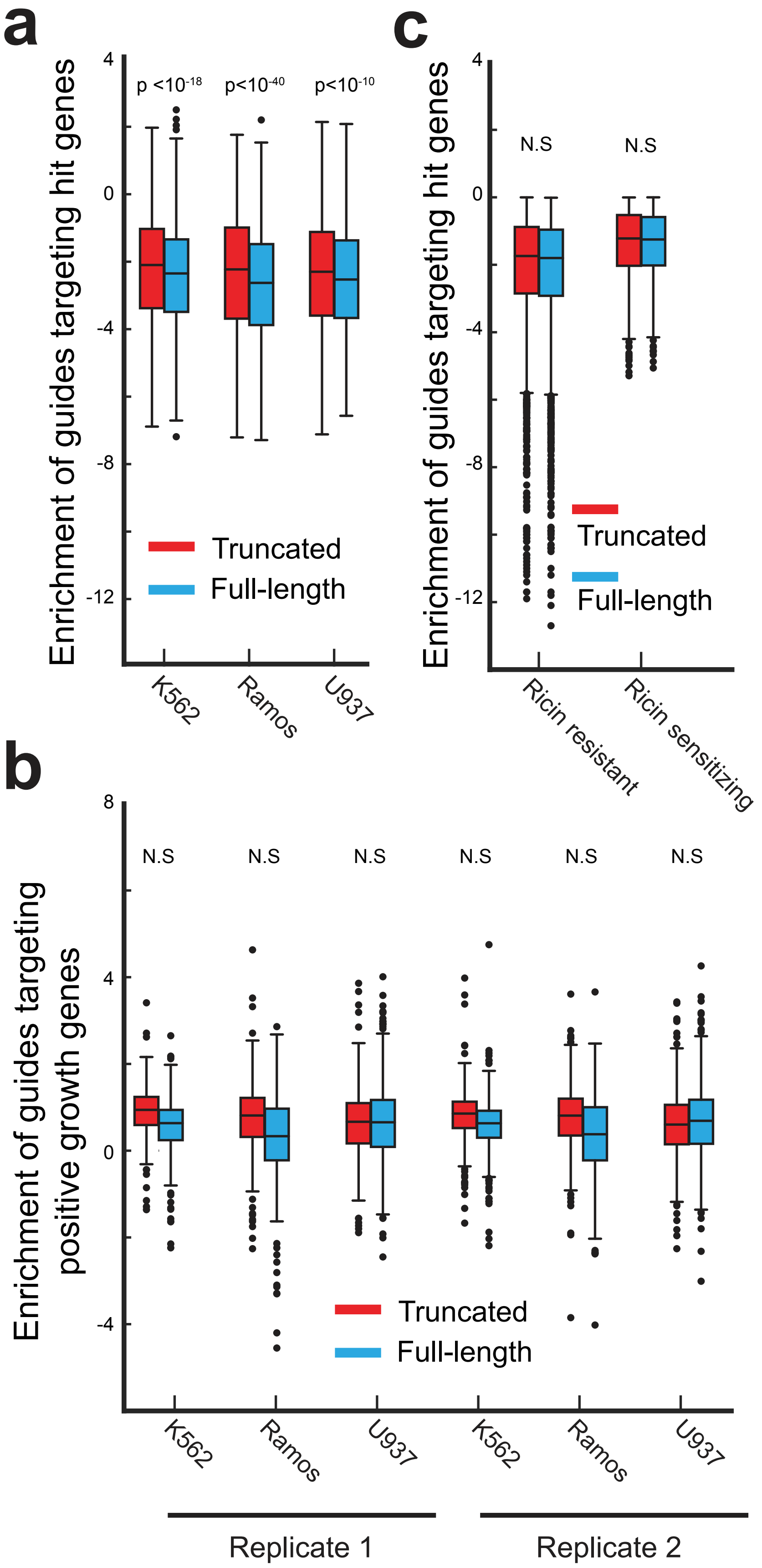

Supplementary Figure 13. Box plot of enrichment scores for guides targeting hit genes at 10% FDR. Box is length of quartile, whiskers represent 1.5x quartile, and dots indicate outliers for guide targeting hit genes in a) growth screens and c) ricin screens. Signs have been flipped for ricin resistant genes for comparison. P-values calculated using a Mann-Whitney test. b) Box plot of enrichment for guides targeting top 100 positive growth genes. Box is length of quartile, whiskers represent 1.5x quartile, and dots indicate outliers. P-values calculated with a single-tailed Mann-Whitney test testing if truncated guides display less activity. N.S indicates non-significance (p > 0.01).
